# Supplementary material for: The Role of Vitamins in Oral Potentially Malignant Disorders and Oral Cancer: A Systematic Review
Source: J Pers Med. 2023 Oct 23;13(10):1520. doi: 10.3390/jpm13101520 (PMC10608573; doi:10.3390/jpm13101520)
Supplement: Supplementary file 1 [file jpm-13-01520-s001.zip › jpm-2676001-supplementary.pdf]

Supplementary

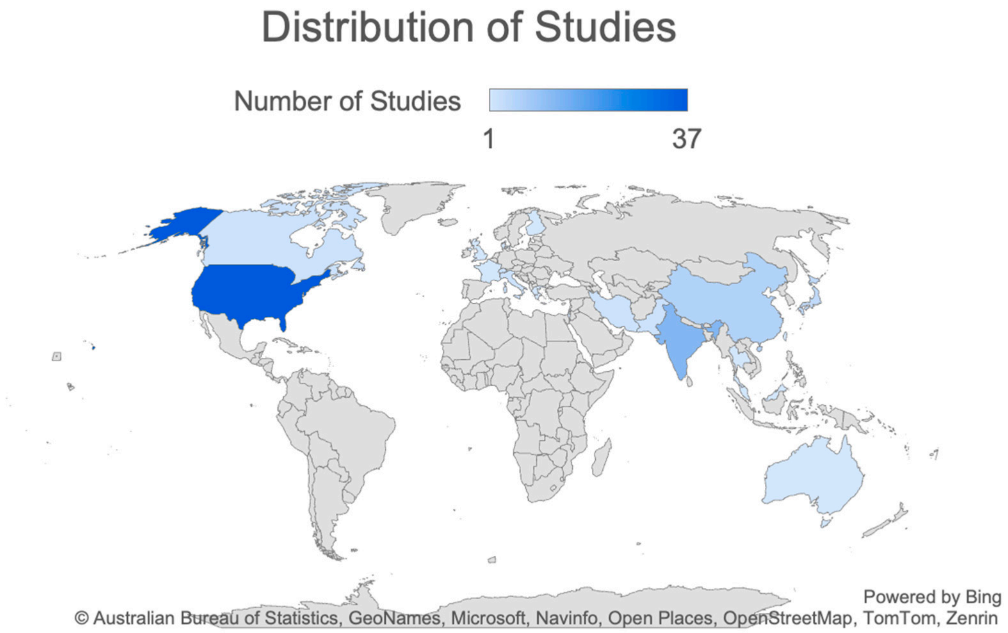

Figure S1: Geographical distribution of the studies that were included in the systematic review.

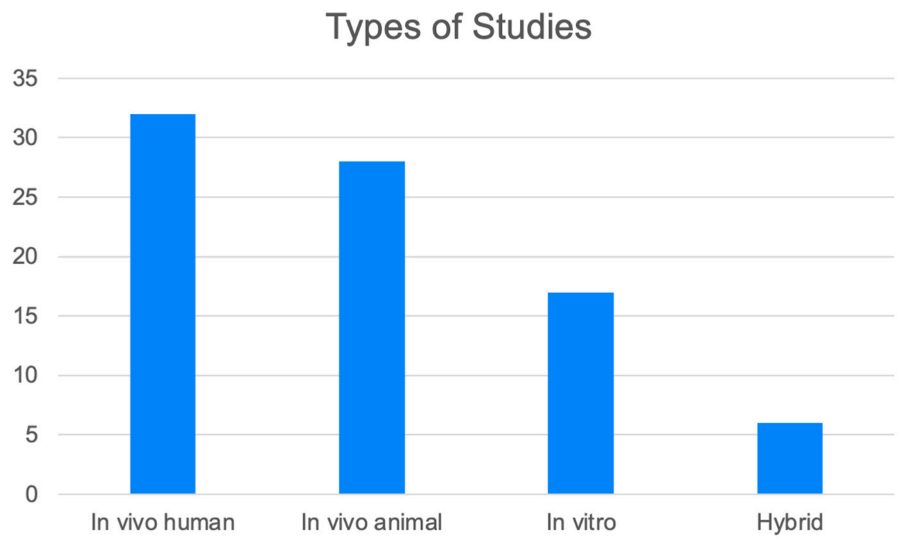

Figure S2: Distribution of the type of studies.

Table S1: List of essential vitamins

Vitamin	Synonym/related terms included in search
Vitamin A	Retinol, retinoid, retinoic acid, retinyl ester, carotenoid, beta-carotene
Vitamin C	Ascorbic acid
Vitamin D	Calciferol
Vitamin E	Tocopherol
Vitamin K	Phylloquinone, menaquinone, phytonadione
Vitamin B1	Thiamine
Vitamin B2	Riboflavin
Vitamin B3	Niacin
Vitamin B5	Pantothenic acid
Vitamin B6	Pyroxidine
Vitamin B7	Biotin
Vitamin B9	Folate
Vitamin B12	Cobalamin

Table S2: OPMDs based on 2020 WHO Consensus (13)

OPMD (abbreviation)	Definition
Oral leukoplakia (OL)	A predominantly white plaque of questionable risk having excluded other known diseases or disorders that carry no increased risk for cancer.
Proliferative verrucous leukoplakia (PVL)	Progressive, persistent, and irreversible disorder characterised by the presence of multiple leukoplakia that frequently becomes warty.
Erythroplakia (EL)	A predominantly fiery red patch that cannot be characterized clinically or pathologically as any other definable disease.
Palatal lesions in reverse smokers (PLRS)	White and/or red patches affecting the hard palate in reverse smokers, frequently stained with nicotine.
Oral submucous fibrosis (SF)	A chronic, insidious disease that affects the oral mucosa, initially resulting in loss of fibro-elasticity of the lamina propria and, as the disease advances, results in fibrosis of the lamina propria and the submucosa of the oral cavity along with epithelial atrophy.
Oral lichen planus (OLP)	A chronic inflammatory disorder of unknown aetiology with characteristic relapses and remissions, displaying white reticular lesions, accompanied or not by atrophic, erosive, and ulcerative and/or plaque-type areas. Lesions are frequently bilaterally symmetrical. Desquamative gingivitis may be a feature.
Dyskeratosis congenita (DC)	A rare cancer-prone inherited bone marrow failure syndrome caused by aberrant telomere biology. It is characterized clinically by the presence of the diagnostic triad of dysplastic nails, lacy reticular skin pigmentation, and oral leukoplakia.
Actinic keratosis/actinic cheilitis (AK)	A disorder that results from sun damage and affects exposed areas of the lips, most commonly the vermilion border of the lower lip, with a variable presentation of atrophic and erosive areas and white plaques.
Oral lupus erythematosus (OLE)	An autoimmune connective tissue disease which may affect the lip and oral cavity, where it presents as an

	erythematous area surrounded by whitish striae, frequently with a target configuration.
Oral lichenoid lesion (OLL)	Oral lesions with lichenoid features but lacking the typical clinical or histopathological appearances of OLP; that is, they may show asymmetry or are reactions to dental restorations or are drug-induced.
Oral Chronic Graft-versus-Host Disease (cGVHD)	Clinical and histopathological presentations similar to oral lichen planus in a patient developing an autoimmune, multi-organ complication after allogeneic hematopoietic cell transplantation.

Table S3: Search string including all keywords and operators. ^: Search string 6 was used in Medline and EBM databases only, as the Web of Science database does not support subject heading-based search.

**Search**	**String**
**1**	("vitamin*" OR "vitamin product*" OR "vitamin deficienc*" OR "vitamin therap*" OR "vitamin A" OR "retinol" OR "retinoid" OR "retinoic acid" OR "retinyl ester" OR "carotenoid" OR "beta-carotene" OR "ascorbic acid" OR "vitamin C" OR "vitamin D" OR "calciferol" OR "vitamin E" OR "tocopherol" OR "vitamin K" OR "phylloquinone" OR "menaquinone" OR "menadione" OR "phytonadione" OR "vitamin B" OR "B group vitamin*" OR "vitamin B1" OR "thiamine" OR "vitamin B2" OR "riboflavin" OR "vitamin B3" OR "niacin" OR "vitamin B5" OR "pantothenic acid" OR "vitamin B6" OR "pyridoxine" OR "vitamin B7" OR "biotin" OR "vitamin B9" OR "folate" OR "vitamin B12" OR "cobalamin")
**2**	("trace element*" OR "mineral*" OR "boron" OR "borate" OR "copper" OR "cobalt" OR "iodine" OR "iron" OR "manganese" OR "molybdenum" OR "zinc" OR "chlorine" OR "fluorine" OR "fluoride" OR "titanium" OR "nickel" OR "selenium" OR "vanadium" OR "bromine" OR "lithium" OR "silicon" OR "tin" OR "stannous" OR "titanium")
**3**	("oral" OR "mouth") adj5 ("premalignan*" OR "precancer" OR "dysplasia" OR "leukoplakia" OR "erythroplakia" OR "lichen planus" OR "submucous fibrosis" OR "lichenoid lesion*" OR "lupus erythematosus" OR "dyskeratosis congenita" OR "chronic graft-versus-host disease" OR "actinic keratosis")
**4**	("oral" OR "mouth") adj5 ("cancer" OR "malignan*" OR "neoplas*")
**5**	("proliferative verrucous leukoplakia" OR "palatal lesions in reverse smokers" OR "actinic cheilitis")
**6^**	exp mouth neoplasms/ OR gingival neoplasms/ OR leukoplakia, oral/ OR lip neoplasms/ OR palatal neoplasms/ OR salivary gland neoplasms/ OR tongue neoplasms/ OR lichen planus, oral/ OR oral submucous fibrosis/
**7**	1 OR 2
**8**	3 OR 4 OR 5
**9**	7 AND 8
**10**	6 AND 9

Table S4: Number of articles and kappa coefficients of record screening by title/abstract.

Screening by Title/Abstract	Number of Articles	Kappa Coefficient
Pilot 1	30	0.93
Pilot 2	50	0.96
Complete	3670	0.96
Vitamins Only	485	0.94

Table S5: In vivo human studies

Author	Year	Type	Additional therapies	Dose	Frequency of administration	Route of Administration	OPMD/ Cancer	Design	Sample size	Risk Factors (#Y, #N, #N/R)	Summary
Bacci et al.	2017	Vit E	N/A	N/R	3/day	Topical	OLP	RCT	33	Smoking (1Y)	Topical tocopherol proved effective in reducing the dimension of OLP lesions but does not decrease discomfort.
Benner et al.	1993	Vit E	N/A	400 IU	2/day	Topical	OL	CT	43	N/A	Administration of alpha-tocopherol resulted in both clinical and histologic responses in premalignant leukoplakia lesions.
Buajeeb et al.	2008	Vit A	N/A	15 mg	Single dose	Oral	OLP	CT	20	N/A	Beta-carotene supplementation significantly reduced micronucleated exfoliated cell frequency in atrophic and erosive OLP, and reduced severity of OLP.
Chitra et al.	2008	Vit E	Radiotherapy	400 IU	1/day	N/R	OC	CT	78	N/R	Glycoconjugate markers of malignant transformation were significantly decreased with Vit E supplementation.
Delavarian et al.	2021	Vit D	N/A	50000 IU	1/week	Oral	OLP	RCT	28	Smoking (3Y)	The severity of the lesions and the levels of IL-6 and TNF-α were reduced in the patients treated with Vit D.
Dhariwal et al.	2010	Vit A	Zinc	25000 IU	1/day	Oral	SF	CR	1	Areca nut (1Y), smoking (1Y)	Vit A and zinc acetate significantly increased mouth opening and epithelial thickness, and decreased the collagen content of lesions.

Epstein et al.	1999	Vit A	N/A	0.05% or 0.01%	4/day	Topical	OL	CR	26	Tobacco use (13Y), alcohol use (7Y)	Approximately 27% of patients had complete clinical remission; recurrence was observed in approximately 40% of patients; 50% decrease in clinical grade of leukoplakia without change in mean histologic grade.
Garewal et al.	1999	Vit A	N/A	60 mg	1/day	Oral	OL	RCT	50	Alcohol (N/R), smoking (N/R)	Beta-carotene was able to decrease leukoplakic lesions observed by decreased thickness of lesions.
Garewal et al.	1990	Vit A	N/A	30 mg	1/day	Oral	OL	CT	24	Alcohol and smoking (9Y), alcohol only (7Y), smoking only (4Y)	Beta-carotene is effective in reversing oral leukoplakia and is well tolerated without any major side effects.
Gunther et al.	1973	Vit A	N/A	30 mg, 0.1%	1/day	Oral, topical	OLP	CT	12	N/R	Discrete and confluent leukoplakia-like papules showed regression after 2-7 weeks after oral administration of Vit A.
Gupta et al.	2019	Vit D	Psychological counselling, topical steroids (N/E)	N/E	N/R	Oral	OLP	PiS	106	Stress only (30Y), Vit D deficiency (46Y), stress and severe Vit D deficiency (30Y)	Reduction of burning sensation and improvement in objective morphological severity with Vit D supplementation.
Johnson et al.	1963	Vit A	N/A	50,000 IU	1/day	Oral	OL	RCT	40	N/R	Reduction in size of lesions and histological improvements in lesions treated with Vit A.
Jolly et al.	1977	Vit A, Vit B, Vit C	N/A	N/R	N/R	N/R	OLP	CT	71	N/R	Complete remission of lesions within 2 months was not seen.
Liede et al.	1998	Vit A, Vit E	N/A	20 mg, 50 mg	1/day	Oral	OL	CT	343	Smoking (343Y)	No evidence to support an effect of cellular beta-carotene concentration on precancerous lesions.

Lin et al.	2011	Vit B	Levamisole	1 mg/cc	N/E	Intramuscular injection	OLP	CT	812	N/R	Gastric parietal cell antibody-positive patients treated with Vit B12 had a reduction in pain or burning sensation caused by the lesion, and healing of the erosive or ulcerative lesion.
Lippman et al.	1993	Vit A	N/A	30 mg	1/day	N/R	OL	CT	53	Alcohol and smoking (31Y), alcohol only (45Y), smoking only (39Y)	Beta-carotene had greater numbers of patients with relapse, and poorer response during maintenance therapy.
Maher et al.	1997	Vit A, Vit B, Vit C, Vit D, Vit E	Minerals	N/E	1/day	Oral	SF	CCS	117	Smoking (13Y)	Beneficial clinical response to SF with multiple micronutrient intervention.
Malaker et al.	1991	Vit A	Cis-retinoic acid	30 mg	N/R	N/R	DYS	PiS	18	Smoking (11Y)	Vit A was able to improve clinical symptoms of mucosal dysplasia such as burning mouth syndrome.
Manas et al.	2022	Vit E	Lycopene, Selenium	400 IU	2/day	Oral	OL	CSS	52	N/R	Vit E significantly reduces the size of oral leukoplakia lesions and the degree of dysplasia.
Mayne et al.	2001	Vit A	N/A	50 mg	1/day	Oral	OC	RCT	264	Smoking (244Y)	No statistically significant evidence found that beta-carotene supplement improved chances of secondary recurrence of head/neck/oral cancer.
Nagao et al.	2015	Vit A, Vit C	N/A	10 mg, 500 mg, 50mg	1/day	Oral	OL	RCT	46	Alcohol (18Y)	One year supplementation with low-dose beta-carotene and Vit C was not significantly effective for clinical remission of OL or to prevent the development of cancer.
Nazeer et al.	2020	Vit D	N/A	60000 IU	N/R	Topical	OLP	CCS	450	N/A	Vit D supplementation decreased lesion size after treatment.

Nilesh et al.	2021	Vit A, Vit E	Lycopene, selenium, zinc sulfate, copper, alpha-lipoic acid	10mg, 10IU	2/day	Oral	SF	CT	46	Areca nut (N/R)	Multidrug therapy was effective in improving burning sensation symptoms and mouth opening in SF patients.
Ong et al.	1982	Vit A	N/A	N/E	N/E	N/A	OC	CCS	6	N/R	Vit A increased retinoic acid-binding protein expression in oral cancer cells.
Razi et al.	2016	Vit E	Betonil	0.5 mL	2/day	Topical	SF	RCT	76	Betel nut (76Y)	Vit E with corticosteroid is more effective than corticosteroid alone at relieving burning sensation (pain) and improving trismus.
Sankaranarayanan et al.	1997	Vit A	N/A	300000 IU, 360 mg	1/week	Oral	OL	RCT	160	Alcohol (72Y), smoking (41Y)	Vit A had a higher percentage of complete regression of OL lesions.
Silverman et al.	1963	Vit A	N/A	75000 U	10/day	Topical	OL	CT	19	N/R	Vit A results in complete/partial remission of OL in most patients.
Silverman et al.	1965	Vit A	N/A	75000 U	8/day	Oral	OL	CT	6	N/R	Varying Vit A supplementation response ranged from complete to no remission of oral leukoplakia; similarly variable response to histological change.
Stich et al.	1988	Vit A	N/A	100 000 IU	2/week	Oral	OL	CT	130	Betel nut (130Y)	Frequency of micronucleated cells was reduced, leukoplakia lesions regressed, and development of new lesions inhibited.
Stich et al.	1988	Vit A	N/A	0.14 mg/kg	1/day	Oral	OL	CT	65	Betel nut (65Y)	The development of new oral leukoplakias was inhibited, and remission of established leukoplakias was induced by the 6-month oral administration of Vit A at a dose of 0.14 mg/kg body weight per day.

Smith	1962	Vit A	N/A	75000-300000 USP	1/day	Oral	OL	CT	417	Tobacco chewing and snuff (102Y),	Early hyperkeratotic lesions showed most improvement; some had achieved complete resolution with Vit A supplementation.
Toma et al.	1992	Vit A	N/A	90 mg	1/day	Oral	OL	CT	18	Alcohol and smoking (12Y), alcohol (2Y), smoking (1Y)	Beta-carotene has "fair efficacy" against OL.
Toma et al.	2003	Vit A	Radiotherapy	75 mg	1/day	Oral	OC	RCT	214	N/A	No statistically significant different effect of Vit A on recurrence and death rate.
Varma et al.	2007	Vit A	N/A	1 IU	2/week	Oral	OL, SF, OLP	CT	24	N/R	Vit A was beneficial in protecting the pre-malignant cases from p53 mutation and bcl2 expression.

Table S6: In vivo animal studies

Author	Year	Vitamin	Additional therapies	Dose	Frequency of administration	Route of Administration	OPMD/ Cancer	Type	Strain	Summary
Bothwell et al.	2015	Vit D	Erlotinib	0.1 µg	3/day	N/R	OC	Mice	SCID	Significant reduction in the degree of dysplasia and suppressed tumour growth with combination treatment of active form of Vit D and erlotinib.
Calhoun et al.	1989	Vit E	13-cis-retinoic acid	2 drops	Single dose	Oral	OC	Hamster	Golden	Reduced ornithine decarboxylase increase upon DMBA insult in early response (8-48 hours); Vit E pre-treatment decreased ornithine carboxylase levels in late response (3-12 days).
Ge et al.	2020	Vit D	N/A	20 nM	7/week	Intraperitoneal injection	OLP	Mice	C57BL/6	Vit D/Vit D receptor signalling accelerates miR-27a/b, non-coding RNAs that regulate inflammatory response, expression in OLP.
Gijare et al.	1990	Vit A	Snuff	5,000 IU	2/week	Intraperitoneal injection	OC	Hamster	Golden	Both beta-carotene and retinoic acid showed a total inhibition of DMBA-induced carcinogenesis in the hamster cheek pouch model.
Harada et al.	1987	Vit C	N/A	1%	N/R	Oral	OL	Hamster	Golden	Incidence of leukoplakic lesions was lower in Vit C supplemented hamsters than those without Vit C.
Huang et al.	2019	Vit D	Cisplatin	30 µg/kg	Single dose	Intraperitoneal injection	OC	Mice	BALB/c	No significant effect on OSCC cell death rate, but when combined with cisplatin, increased cell death in vitro and in vivo (animal).
Kandarkar et al.	1991	Vit C	N/A	15 mg	3/week	Topical	OC	Rat	Sprague Dawley	Mice treated with topical Vit C showed delayed progression.
Kandarkar et al.	1990	Vit A	N/A	N/E	N/R	N/R	OC	Hamster	Golden	Decreased progression of lesion size and dysplastic changes with supplementation.

Meier et al.	2007	Vit D	N/A	0.25 µg/kg	3/week	Intraperitoneal injection	OC	Hamster	Golden	Vit D delays carcinogenesis.
Odukoya et al.	1984	Vit E	N/A	47.5 mg	3/week	Topical	OL, OC	Hamster	Golden	Vit E slowed tumour formation, and tumours were smaller and fewer with better cellular differentiation and less invasion.
Polliack et al.	1971	Vit A	N/A	1.7 million IU	3/week	Topical	OL	Hamster	Golden	Vit A caused leukoplakia to progress to squamous cell carcinoma in the hamster buccal pouch model.
Potdar et al.	1992	Vit C	DMBA	500 µg	N/A	Topical	OC	Hamster	Golden	Vit C was able to restrict growth and invasion of carcinomas into sub-epithelium.
Rowe et al.	1959	Vit A	N/A	400 IU	2/week	Oral	OC	Hamster	Golden	Vit A deficiency promotes epithelial tumour production in carcinogen-treated cheek pouch.
Rubin et al.	1973	Vit D	N/A	1.6 mg	3/week	Topical	OC	Hamster	Golden	Vit D (D2 and D3) reduced incidence of DMBA-induced tumorigenesis.
Salley et al.	1962	Vit B	N/A	0.400 mg/100 gm	1/day	N/R	OC	Hamster	Pee-Dee	Latent period for tumor induction was significantly shortened in the group maintained on a low-thiamine diet.
Sawant et al.	2000	Vit C, Vit E	N/A	50 µg	3/week	Topical	OC	Hamster	Golden	Vit C and E, and in combination, delay tumour induction, reduce the size and number of tumours, inhibit cell proliferation, restrict progression and invasion, and appear to prevent dysplasia.
Schwartz et al.	1990	Vit A	N/A	N/A	N/R	Topical	OC	Hamster	Golden	Beta-carotene administration resulted in prevention, inhibition, and regression of OSCC in the hamster tumour model.
Schwartz et al.	1988	Vit A	Canthaxanthin	250 µg	2/week	Submucosal injection	OC	Hamster	Golden	A significant regression of established epidermoid carcinomas of hamster buccal pouch was effected by the local injection of beta-carotene and canthaxanthin but not by 13-cis-retinoic acid.
Schwartz et al.	1993	Vit C	N/A	1 mg	3/week	Oral	OC	Hamster	Golden	Vit C had significantly larger tumours and the number of gross tumours were slightly increased in the hamster buccal pouch model.

Schwartz et al.	1989	Vit A	N/A		350 μg	2/week	Submucosal injection	OC	Hamster	Golden	Cyanobacteria extract and beta-carotene significantly increased rate of gross reduction of tumour.
Schwartz et al.	1986	Vit A	N/A		N/E	2/week	Topical	OC	Hamster	Golden	Topical and subcutaneous injection of beta-carotene significantly reduced lesion count.
Shklar	1982	Vit E	N/A		10 mg	2/week	Topical	OC	Hamster	Golden	Inhibition of oral mucosal carcinogenesis in hamsters by injection of Vit E1.
Shklar et al.	1987	Vit E	N/A		250 μg	2/week	Submucosal injection	OC	Hamster	Golden	Regression of established, chemically induced tumours was effected by local injection of Vit E.
Suda et al.	1987	Vit A	N/A		25 mg/kg	3/week	Topical	OC	Hamster	Golden	Animals that received beta-carotene treatment had lower number of tumours, reduced size of tumours, and more well-differentiated cancer cells which were less invasive.
Suda et al.	1986	Vit A	N/A		0.62 mg, 190 ng/ml	3/week	Topical	OC	Hamster	Golden	Animals that received beta-carotene treatment had lower number of tumours, reduced size of tumours, and more well-differentiated cancer cells which were less invasive; beta-carotene offered chemoprotection.
Tanaka et al.	1994	Vit A	N/A		500 ppm	N/R	Oral (diet)	OC	Rat	F344	Beta-carotene in the diet inhibited rat oral carcinogenesis that was initiated with 4-nitroquinoline 1-oxide.
Trickler et al.	1987	Vit E	N/A		10mg	2/week	Oral	OC	Hamster	Golden	Vit E prevented carcinogenic action in the hamster buccal pouch model.
Verma et al.	2020	Vit D	N/A		25 IU, 100 IU, 10 000 IU	N/R	Oral	OC	Mice	C57BL	High-grade dysplasia and OSCC was lower in mice on 100 IU vitamin D diets; mice on the 10 000 IU showed highest incidence of OSCC
Weerapradist et al.	1982	Vit E	N/A		7 IU	2/week	Oral	OC	Hamster	Golden	Vit E is capable of delaying tumour formation or retarding carcinogenesis.

Table S7: In vitro studies

Author	Year	Vitamin	Additional therapies	Dose	Frequency of administration	Route of Administration	OPMD/ Cancer	Cell type	Cell name	Summary
Abe et al.	1998	Vit D	N/A	10^-7 M	Single dose	N/A	OC	Cancer	HSC-3 cells	Vit D and 9-cis-retinoic acid suppressed parathyroid hormone-related protein production and its mRNA expression in the human OSCC cell line.
Dalirsani et al.	2012	Vit D	5-fluorouracil, 13-cis retinoic acid	10 µmol and 20 µmol	Single dose	N/A	OC	Cancer	C152	Vitamin D decreases SCC proliferation.
Elattar et al.	1999	Vit E	N/A	0.001-154 µmol/L	N/R	N/A	OC	Cancer	N/A	Physiological concentrations of Vit E succinate (VES) enhanced cell growth (0.001-50 µmol/L); pharmacological concentrations of VES inhibited cell growth (100 and 154 µmol/L)
Ge et al.	2020	Vit D	N/A	300 ng/kg	7/week	Intraperitoneal injection	OLP	Hybrid	Oral epithelial cell	Vit D/Vit D receptor signalling accelerates miR-27a/b, non-coding RNAs that regulate inflammatory response, expression in OLP.
Ge et al.	2022	Vit D	N/A	20 nM	Single dose	N/A	OLP	Hybrid	Oral epithelial cell	Vit D lowers STING (Stimulator of interferon genes) and IFNβ overexpression in OLP.
Huang et al.	2019	Vit D	Cisplatin	30 nM	Single dose	N/A	OC	Cancer	CAL-27 SCC-9	No significant effect on OSCC cell death rate, but when combined with cisplatin, increased cell death in vitro and in vivo (animal).
Jin et al.	2020	Vit D	N/A	30 nM	Single dose	N/A	OC	Cancer	N/A	Vit D inhibited OSCC growth.

Kingsley et al.	2013	Vit D	Genistein, Daidzein Glycitein	10 nmol, 50 nmol, 150 nmol	Single dose	N/A	OC	Cancer	CAL-27, SCC-25	Increased concentration of Vit D3 results in more robust inhibition of SCC proliferation, and coadministration of Vit D3 and soy isoflavones further enhances their anti-tumour effect.
Liede et al.	1998	Vit A, Vit E	N/A	N/A	N/A	Oral	OL	Hybrid	N/A	No evidence to support an effect of cellular beta-carotene concentration on precancerous lesions.
McCabe et al.	2010	Vit B	N/A	0, 10, 50, 100, 200, 400 and 1000 µg/mL	Single dose	N/A	OC	Cancer	CAL27, SCC25	Vit B9 increased OSCC proliferation (dose-dependent).
Moody et al.	2012	Vit B	N/A	N/E	N/E	N/A	OC	Cancer	CAL27, SCC15, SCC25, HGF-1	Antimetabolites of Vit B can be alternative treatments for resistant tumours.
Odukoya et al.	1986	Vit E	N/A	0.1, 1, 10, 100 µM	1/5 days	N/A	OC	Cancer	HCPC-1	Vit E at low doses increased OSCC growth (max at 10 µM); at high doses it decreased growth (100 µM).
Okayasu et al.	2001	Vit K	N/A	N/E	Single dose	N/A	OC	Cancer	HSC-2, HSG, HL-60, HGF	Vit K3 is a more cytotoxic and more efficient O_2 scavenger than Vit K1 and K2.
Ong et al.	1982	Vit A	N/A	N/E	N/E	N/A	OC	Cancer	N/A	Vit A increased retinoic acid-binding protein expression in oral cancer cells.
Schwartz et al.	1989	Vit A	N/A	2-60 µg/mL	Single dose	N/A	OC	Cancer	HCPC-1	Cyanobacteria extract and beta-carotene reversed peritoneal exudate cell cytotoxicity to HCPC-1 tumour cells.
Schwartz et al.	1986	Vit A	N/A	N/E	N/A	Subcutaneous injection	OC	Cancer	HCPC-1	Tumour necrosis-positive macrophages from animals treated with beta-carotene were upregulated.
Sundaram et al.	2014	Vit D	N/A	10^-8 M	Single dose	N/A	OC	Cancer	SCC1, SCC11B, SCC14a	Vit D analogues could be potential therapeutic agents to control OSCC tumour progression.

Suresh et al.	2013	Vit K	N/A	1-100 μM	Single dose	N/A	OC	Cancer	SAS	Vit K3 is more cytotoxic to oral cancer cells but not non-tumorigenic cells, while also exhibiting anti-neoplastic and antimigratory effects, effectively blocking epithelial to mesenchymal transition in oral cancer cells.
Toma et al.	1991	Vit A	N/A	10 μM	Single dose	N/A	OL, OC	Dysplasia, cancer	KB, SCC-25	Beta-carotene was able to reduce clonogenic activity, even if it does not seem to influence cell proliferation, and it has a protective effect against genotoxic damage.
Zhao et al.	2018	Vit D	N/A	N/E	Single dose	N/A	OLP	Dysplastic	HaCat cells	Vit D/Vit D receptor plays a protective role, stopping or delaying OLP development.
Zhang et al.	2007	Vit E	13-cis-retinoic acid, interferon -alpha2A	15 μM	Single dose	N/A	OC	Cancer	SQCCY1	Combination treatment of 13-cis-retinoic acid, interferon-alpha2A and alpha-tocopherol (Vit E) had a cooperative inhibitory effect on the growth of oral cancer cells.
Zhao et al.	2019	Vit D	N/A	20nM	Single dose	N/A	OLP	Dysplastic	N/A	Vit D/Vit D receptor signalling suppressed miR-802 expression in LPS-treated or activated CD4+ T cell-stimulated human oral keratinocytes by blocking NF-kB pathways inhibiting OLP apoptosis.
Zulkapli et al.	2017	Vit E	Cisplatin	2.5 ± 0.42 μg/mL	N/R	N/R	OC	Cancer	ORL-48 cells	Alpha-Tocopherol was reported effective in enhancing the tumour growth inhibition activity of cisplatin.

Table S8: OHAT risk of bias assessment criteria

Selection Bias:

- Was administered dose or exposure level adequately randomized?
- Was allocation to study groups adequately concealed?

Performance Bias:

- Were experimental conditions identical across study groups?
- Were the research personnel and human subjects blinded to the study group during the study?

Attrition/Exclusion Bias:

- Were outcome data complete without attrition or exclusion from analysis?

Detection Bias:

- Can we be confident in the exposure characterization?
- Can we be confident in the outcome assessment?

Selective Reporting Bias:

- Were all measured outcomes reported?
